# Language learning environment: Spatial perspectives on SLA

**DOI:** 10.3389/fpsyg.2022.958104

**Published:** 2022-09-07

**Authors:** Fang Wang, Jun Zhang, Zaibo Long

**Affiliations:** ^1^School of Foreign Languages, Jingzhou University, Jingzhou, China; ^2^School of Foreign Languages, Wuhan University of Science and Technology, Wuhan, China

**Keywords:** language learning environment, spatial perspectives, second language acquisition, social turn, multilingual turn, ecological view

Before the1970s, second language acquisition (SLA) research was mainly focused on language forms and structures. After that period, SLA researchers turned their spotlight and began to pay attention to the process of language learning. As a result, we knew about both what second language learners learn and how they learn it. However, the research on language learning setting, that is, the language learning environment, was often reduced to research on person-to-person interaction, neglecting the relationship between learners and the language learning environment. With the development of global society and economy, acceleration of globalization, and mobility of the world population, the language learning environment has become increasingly diverse and complex. Researchers on SLA call for a new and comprehensive spatial perspective to grasp the all-around interaction between second language learners and the world in which they lead their lives. The book under review (Benson, 2021) comes out under such a circumstance with spatial perspectives on SLA. In the book, the author explains what roles the language learning environment plays in SLA.

The monograph consists of six chapters. Chapter one explains the reason why SLA researchers should study the language learning environment in space. The reason is that population movements associated with internal and external migration and social mobility, such as the circuits of commodity production and distribution, create much space in which the language learning environment becomes diverse and uneven. As the author claims, with a spatial perspective we can fully understand the interactions between language learners and the world or environments.

In Chapter two, by introducing the brief history of Critical Spatial Theory, the author intends to convey to readers the key idea in the theory: the social production of space, which views space as the product of the social relations of its era. Based on this idea, the author contends that the production of languages is an integral aspect of the production of space on a global scale. This view helps readers to understand how social mobility shapes language learning environments. From this perspective, local environments in which languages are learned are outcomes of the production of space under the influence of mobility. The author reiterates the key view in the chapter that the global mobility of physical objects (people, goods, and information) produce space.

In Chapter three, the author introduces the mainstream views held by scholars of modern linguistics: languages are conceptualized as objects in the space of the world. They hold that the spatiality of language is given by providing correct writing systems and standardized rules for various languages. Influenced by the mainstream conception of language from modern linguistics, SLA tends to regard languages as self-contained objects in space. However, this view neglects the relationship between language and learning environments. The author holds that the distribution and circulation of languages and their varieties in geographical space produce the spatiality of language, which clears the ground for an environment or object-as-space view of language in Chapter four.

In the fourth chapter, the author develops an alternative “objects-as-space” view and introduces three concepts to illustrate the spatiality of language: flat ontology, assemblage, and mobility. The concept of flat ontology helps explain what language is and how it comes into being in the world. From an assemblage perspective, the author develops the idea that everything is an object, and so are human beings and languages. Language (the non-physical object) interacts with various physical objects, forming the language-bearing assemblage. This assemblage is the form of the spatiality of language. The mobility theory explains how language-bearing assemblages develop or change on a global scale because of social mobility, emphasizing the importance of the learning environment in SLA. In brief, the author outlines how language makes its way into physical space in the form of assemblages or assemblages of assemblages.

Chapter five is centered on the language learning environment. First, the author explains the relationship between language learners and their learning environment from an ecological view: the learner is a part of the language learning environment, and second language learning is a spatial interaction between learners and the semiotic resources provided by the environment. Then, the author discusses the language environment from two different perspectives. From an “individual” perspective, environments are assembled by individuals from spatial resources available; from an “areal” view, environments are shaped both by local circumstances and global circulations of language-bearing assemblages. Meanwhile, the author also discusses other language environments such as the multilingual city and online resources. What the author hopes to develop in this chapter is an environmental perspective that grounds the contexts of language learning in space, which helps readers think about language learning from a spatial perspective.

Chapter six contains three parts. In the first part, it outlines the work of a number of researchers from sociolinguistics and the contribution they made to spatial theory. In the second part, the author wants to tell us how the idea of a language learning environment emerges from his research. The last part discusses the four main research routes (areal studies, studies on individuals learning in settings, studies on construction of individual learning environments, and design-based studies) that might influence SLA research on language learning environments and some research methods.

The book is in alignment with several recent “turns” occurring in the field of SLA, such as the “social turn” (Block, 2003) and “multilingual turn” (May, 2014), both of which bring to the fore the social aspects involved in language learning. While the first “turn” accentuates the role of social context in shaping and influencing learner cognition, the latter highlights how the physical multilingual world remolds language learning experiences, and how the mental multilingual world helps raise learners' language awareness and facilitates their successful construction of an identity of legitimate multi-language users. The book under review joins up the two “turns” in acknowledging that language learners are persons-in-context with organic interaction with the social learning environment (Ushioda, 2021). This view has research and pedagogical implications. From the point of doing research, spatial perspectives advocated in the book afford huge research potentials for both language teachers and (instructed) SLA researchers to conduct situated investigations. They will be encouraged to explore the complex and dynamic nexus between language learners and their surrounding environment (not restricted to people) that plays a part in their learning experience. This exploration will empower them to gain an insightful understanding of how individuals and learning contexts are coupled with each other, how they evolve together, and how they constitute an ecosystem that generates affordances, facilitating, or constraining language learning. Pedagogically speaking, the spatial perspectives impel language teachers to hold an ecological view of language teaching and learning, directing their attention to the individuality of each language learner and their complex, dynamic, and multifaceted relationality with other learners and the physical realities, rather than solely focusing on abstract systems (e.g., development of cognitive aspect). This holistic view allows language teachers to take measures to address concrete questions emerging in and out of classrooms and stimulates them to design and experiment with pedagogical interventions that help to create a favorable environment for language learning. This view also invites language teachers to take learner agency into account (Xu and Long, 2020) and to design pedagogical activities that enable learners to take agentive actions to adapt to their environments as they actively create and seek their language learning opportunities, making the world their own (Bronfenbrenner, 1979). In turn, teachers' agency-enabling activities will ultimately help learners autonomously access a range of resources in this new affordance-rich era to improve their language skills and thus become more independent in their pathway to lifelong learning.

Besides the above benefits, the book is also theoretically and methodologically friendly. In illustrating the role of space in SLA, the author invokes a plethora of theories from disciplines such as sociology, sociolinguistics, and psychology. This orientation helps readers to build a transdisciplinary framework to capture the multi-layered nature of SLA (The Douglas Fir Group, 2016) and to acquire a nuanced understanding of how language learning is influenced by multiple scales of environmental factors. Methodologically, the author proposes several specific methods that are especially apt to investigate the language learning environment. These methods, together with the theoretical foundation laid by the book, will help researchers and language teaching practitioners to further delve into the complex interplay between context and language learners.

While the author has made a great contribution to our understanding of the role that the physical environment plays in SLA, future researchers will find it worthwhile to explore how the virtual environment influences SLA processes. This effort will allow second language teachers and learners to have an even more in-depth understanding of SLA, especially in these years of the pandemic when language learning in virtual spaces is becoming a norm. Only when a complete picture of the SLA environment is gained can teachers and learners take full advantage of the affordances (van Lier, 2004) provided by the environment.

All in all, the book is an invaluable contribution to the field of SLA and is worthy of recommending to language teaching practitioners, researchers, and postgraduates in the field of applied linguistics and second language acquisition.

## Author contributions

FW wrote the initial draft. JZ proof-read the draft and provided several rounds of critical feedback to the drafts on linguistic aspects in the process of writing. ZL provided the last round of feedback on structural and disciplinary aspects. The final draft is the result of FW, JZ, and ZL's collective effort. All authors contributed to the article and approved the submitted version.

## Funding

This study was supported by the Philosophical and Social Science Research Project of the Department of Education of Hubei Province (Grant No. 21Q024).

## Conflict of interest

The authors declare that the research was conducted in the absence of any commercial or financial relationships that could be construed as a potential conflict of interest.

## Publisher's note

All claims expressed in this article are solely those of the authors and do not necessarily represent those of their affiliated organizations, or those of the publisher, the editors and the reviewers. Any product that may be evaluated in this article, or claim that may be made by its manufacturer, is not guaranteed or endorsed by the publisher.
